# Hypomethylating agent monotherapy in core binding factor acute myeloid leukemia: a French multicentric retrospective study

**DOI:** 10.1007/s00277-024-05623-0

**Published:** 2024-01-26

**Authors:** Ludovic Gabellier, Pierre Peterlin, Sylvain Thepot, Yosr Hicheri, Franciane Paul, Maria Pilar Gallego-Hernanz, Sarah Bertoli, Pascal Turlure, Arnaud Pigneux, Romain Guieze, Marlène Ochmann, Jean-Valère Malfuson, Thomas Cluzeau, Xavier Thomas, Emmanuelle Tavernier, Eric Jourdan, Sarah Bonnet, Jean-Jacques Tudesq, Emmanuel Raffoux

**Affiliations:** 1 Département d’Hématologie Clinique, CHU Montpellier, Université Montpellier-Nîmes, 80, Avenue Augustin Fliche, 34090 Montpellier, France; 2 Département d’Hématologie Clinique, CHU Nantes, Université de Nantes, Nantes, France; 3 Département d’Hématologie Clinique, CHU Angers, Université d’Angers, Angers, France; 4 Département d’Hématologie Clinique, Institut Paoli-Calmettes, Marseille, France; 5 Département d’Hématologie Clinique, CHU Poitiers, Université de Poitiers, Poitiers, France; 6 Service d’Hématologie Clinique, CHU Toulouse, Institut Universitaire du Cancer de Toulouse - Oncopôle, Université Toulouse III - Paul Sabatier, Toulouse, France; 7 Département d’Hématologie Clinique, CHU Limoges, Université de Limoges, Limoges, France; 8 Département d’Hématologie Clinique, CHU Bordeaux, Université de Bordeaux, Bordeaux, France; 9 Département d’Hématologie Clinique, CHU Clermont-Ferrand, Université de Clermont-Ferrand, Clermont-Ferrand, France; 10 Département d’Hématologie Clinique, Orléans, Orléans, CH France; 11 Département d’Hématologie Clinique, Hôpital d’instruction Des Armées, Percy, France; 12 Département d’Hématologie Clinique, CHU Nice, Université de Nice, Nice, France; 13 Département d’Hématologie Clinique, Hospices Civils de Lyon, CHU Lyon, Université de Lyon, Lyon, France; 14 Département d’Hématologie Clinique, Institut de Cancérologie Lucien Neuwirth, Université de Saint-Etienne, Saint-Etienne, France; 15 Département d’Hématologie Clinique, CHU Nîmes, Université de Montpellier-Nîmes, Nîmes, France; 16 Département d’Hématologie Clinique Adultes, Hôpital Saint-Louis, APHP, Université Paris Diderot, Paris, France

**Keywords:** Acute myeloid leukemia, Core binding factor, Hypomethylating agents, Azacitidine, Decitabine

## Abstract

Very few data are available about hypomethylating agent (HMA) efficiency in core binding factor acute myeloid leukemias (CBF-AML). Our main objective was to evaluate the efficacy and safety of HMA in the specific subset of CBF-AML. Here, we report the results of a multicenter retrospective French study about efficacy of HMA monotherapy, used frontline or for R/R CBF-AML. Forty-nine patients were included, and received a median of 5 courses of azacitidine (*n* = 46) or decitabine (*n* = 3). ORR was 49% for the whole cohort with a median time to response of 112 days. After a median follow-up of 72.3 months, median OS for the total cohort was 10.6 months. In multivariate analysis, hematological relapse of CBF-AML at HMA initiation was significantly associated with a poorer OS (HR: 2.13; 95%CI: 1.04–4.36; p = 0.038). Responders had a significantly improved OS (1-year OS: 75%) compared to non-responders (1-year OS: 15.3%; p < 0.0001). Hematological improvement occurred for respectively 28%, 33% and 48% for patients who were red blood cell or platelet transfusion-dependent, or who experienced grade 3/4 neutropenia at HMA initiation. Adverse events were consistent with the known safety profile of HMA. Our study highlights that HMA is a well-tolerated therapeutic option with moderate clinical activity for R/R CBF-AML and for patients who cannot handle intensive chemotherapy.

**Supplementary Information:**

The online version contains supplementary material available at 10.1007/s00277-024-05623-0.

## Introduction

Acute myeloid leukemia (AML) is a type of cancer derived from an oligoclonal proliferation of undifferentiated hematopoietic myeloid precursors[1]. Core-Binding Factor AML (CBF-AML) account for about 10–15% of adult AML[2, 3], and are graded in the favorable risk group of European LeukemiaNet (ELN) classification[4]. CBF-AML are characterized by either the chromosomal translocation t(8;21)(q22;q22.1) or rearrangement inv(16)(p13.1q22)/t(16;16)(p13.1;q22), respectively leading to the translation of aberrant fusion proteins RUNX1::RUNX1T1 and CBFB::MYH11[5–7]. Where, under normal conditions, RUNX1 and CBFB form a heterodimeric protein complex of transcription factors involved in normal myeloid differentiation, in CBF-AML, those fusion transcripts induce, through aberrant epigenetic mechanisms, the silencing of genes involved in normal hematopoiesis[8, 9], and drive the differentiation blockade in CBF-AML[10, 11].

When a cytarabine-based chemotherapy is used as first-line treatment, reported first complete remission rate is about 90%, even in elderly[12–14]. Nonetheless, 5-year relapse rate is 30–40% and 5-year overall survival (OS) is 60–75%, suggesting the heterogeneity of the disease[2]. Several prognostic factors are associated with CBF-AML outcomes, such as age, white blood cell (WBC) count at diagnosis, cooperative tyrosine-kinase mutations (*KIT*, *FLT3*, *N/KRAS*) or clonal architecture[15–22]. Measurable residual disease (MRD) is also a strong prognosis factor to identify patients with a high risk of relapse[23]. An early MRD reduction ≥ 3 log has been shown to significantly lower the incidence of relapse in multivariate analysis[24], whereas MRD persistence in blood at the end of treatment is associated with higher levels of relapse in CBF-AML with t(8;21)[25].

In retrospective studies about outcomes of relapsed / refractory (R/R) CBF-AML, reported second complete remission rate remains high (> 75%) in patients treated with high-dose chemotherapy, gemtuzumab ozogamicin (GO) and/or allogeneic stem cell transplant (ASCT), but OS seems shorter than for patients in CR1, especially in the elderly, or when the CR1 duration was short[26, 27]. Moreover, even if CBF-AML are classified as favorable prognosis AML, relapses after intensive chemotherapy remain a major cause of death[26], and a significant proportion of patients (elderly, patients with poor performance status) may not handle intensive chemotherapy toxicities. In these situations, use of hypomethylating agents (HMA), such as azacitidine or decitabine, might be a therapeutic option. HMA has now become the standard treatment for frail patients with high-risk myelodysplastic syndromes and AML, alone or in combination with other therapies such as venetoclax[28–32]. Nevertheless, given that these studies excluded or did not specifically analyze patients with CBF-AML, and that the proportion of CBF-AML decreases with age[33, 34], very few data were published about HMA efficacy in the specific CBF-AML subset.

Here, we report the results of a multicenter retrospective French study about effectiveness and safety of HMA as monotherapy, used in frontline or for R/R CBF-AML.

## Methods

### Patients and data collection

We retrospectively screened patients aged ≥ 18 years receiving HMA as monotherapy for frontline treatment or for R/R CBF-AML (cytologic relapse or molecular relapse/progression), diagnosed according to the 2016 World Health Organization classification[35], in 17 French centers between January 2008 and December 2019. CBF-AML associated translocations and/or corresponding fusion transcripts had to be confirmed by conventional karyotype, fluorescence in situ hybridization or by RT-qPCR. Patients with CBF-AML in complete response who received HMA for preventive treatment after ASCT for were excluded. No other exclusion criterion was applied.

Clinical, biological and treatment-related data were retrospectively gathered from available medical reports. When available, molecular MRD levels performed in peripheral blood and/or bone marrow by RT-qPCR were collected. Declared hematological and non-hematological adverse events were classified according to Common Terminology Criteria for Adverse Events (CTCAE) version 5.0[36].

According to French laws and national guidelines, retrospective studies using data from medical charts only require a declaration to the “*Commission Nationale Informatique & Libertés*” (CNIL, declaration number 2761316)[37]. There was therefore no requirement for a declaration to an ethics committee.

### End points and statistical analysis

Primary end point was overall survival (OS). Secondary end points included (i) event-free survival (EFS), (ii) overall response rate (ORR), (iii) transfusion independence rate and hematological improvement for neutrophils, and (iv) description of reported adverse events during HMA courses.

Data are described as median and ranges for quantitative variables, and frequency and percentages for qualitative variables. Characteristics of subgroups were compared with nonparametric tests (Fisher exact test for qualitative variables, Wilcoxon test for quantitative variables). Survival analysis were assessed using Kaplan–Meier method. OS was defined as time from the first HMA injection to death resulting of any cause. Alive patients were censored at the end of follow-up. The effect of pre-therapeutic parameters on OS was tested with univariate and multivariate Cox model. EFS was defined as time between HMA initiation and any event (treatment failure, progressive disease, hematologic relapse or death). Statistical significance in OS differences between groups was determined by the log-rank test.

When possible, CBF-AML status and response were assessed retrospectively according to 2017 and 2018 ELN recommendations for all HMA courses[38, 39]. ORR was defined by complete remission with undetectable MRD (CR_MRD-_), complete remission (CR), complete remission with incomplete hematologic recovery (CRi) or partial remission (PR). Treatment failure was defined as a death of indeterminate cause occurring within the 7 days after HMA initiation. Transfusion independence, for red blood cells (RBC) or for platelets, was defined as no transfusion for more than two consecutive HMA courses for transfusion-dependent patients at HMA initiation[40, 41]. Hematological improvement for neutrophils was defined by an at-least 100% increase and an absolute increase > 0.5 G/L of the absolute neutrophil count, occurring at any point during HMA treatment for patients who had a grade 3 or 4 neutropenia at HMA beginning[41].

All statistical tests were two-tails and Alpha-risk was fixed at 5%. Analyses were performed using R.4.1.1. for Mac (R Core Team 2021, R Foundation for Statistical Computing, Vienna, Austria).

## Results

### Patients and CBF-AML characteristics at HMA initiation

We identified 81 patients who received at least one dose of azacitidine or decitabine for CBF-AML treatment. Thirteen patients were not included due to missing data. Respectively 8 and 11 patients were excluded for receiving HMA for post-ASCT prophylactic therapy, and for receiving concomitant anti-leukemic therapies (tyrosine-kinase inhibitors, venetoclax, gemtuzumab-ozogamicin or donor lymphocyte infusions). Therefore, 49 patients were included for final analysis. At HMA onset, median age was 63 years (range 23–86) for the whole cohort, with a *sex ratio* (female/male) of 1.04. Twelve patients (24%) had secondary AML, mostly due to prior chemotherapy and/or radiotherapy for solid tumor or lymphoid malignancy. Eight patients (16%) experienced an extra-medullar and/or a central nervous system involvement of CBF-AML. Patients and CBF-AML characteristics at HMA initiation are summarized in Table 1.

**Table 1:** Patients and CBF-AML characteristics at HMA initiation

	Total cohort (*n* = 49)	Therapeutic line		
		First-line (*n* = 20)	Second-line or more (*n* = 29)	*p*-value
Age (years)	63 [23—86]	74.5 [33—86]	52 [23—80]	< 0.001
Gender (female)	25 (51%)	9 (45%)	16 (55%)	ns
Performance status:				0.04
• PS 0 or 1 • PS 2 or 3	37 (76%) 12 (24%)	12 (60%) 8 (40%)	25 (86%) 4 (14%)	
CBF-AML subtype:				ns
• t(8;21) and/or *RUNX1*::*RUNX1T1* transcript • inv(16) or t(16;16) and/or *CBFB*::*MYH11* transcript	21 (43%) 28 (57%)	8 (40%) 12 (60%)	13 (45%) 16 (55%)	
Secondary CBF-AML	12 (24%)	7 (35%)	5 (17%)	ns
Extra-medullar and/or CNS involvement	8 (16%)	1 (5%)	7 (24%)	ns
Biological data:				
• Bone marrow blasts (%) • Hemoglobin (g/L) • White blood cells (G/L) • Neutrophils (G/L) • Platelets (G/L)	29 [1—88] 104 [61—142] 4.0 [0.8—190.3] 1.4 [0.0—22.0] 66 [4—227]	32.5 [17—81] 98.5 [64—126] 5.7 [1.1—190.3] 1.3 [0—11.0] 46 [7—227]	18.5 [1—88] 107 [61—142] 3.6 [0.8—41.8] 1.9 [0.2—22.0] 74 [4—226]	0.02 ns 0.01 ns ns
Additional cytogenetic abnormalities	24 (49%)	9 (45%)	15 (52%)	ns
Tyrosine-kinase mutations (mutated / tested)		-	-	*not tested*
• FLT3-TKD • KIT • NRAS	3/22 1/9 1/5			
Number of prior therapeutic lines	-	-	2 [1–4]	*not tested*
Description of prior therapeutic lines:	-	-		*not tested*
• Intensive chemotherapy* without stem cell transplant • Intensive chemotherapy* and autologous stem cell transplant • Intensive chemotherapy* and allogeneic stem cell transplant			18 (62%) 1 (3%) 10 (34%)	
CBF-AML status at HMA initiation: • Diagnosis	-	20 (41%)	-	*not tested*
• Cytologic relapse • Molecular relapse or progression		- -	24 (49%) 5 (10%)	

Data are median [min–max] or number (%). Results are presented for the whole cohort (*n* = 49), and according to therapeutic line of HMA.

* Including cytarabine, anthracyclines, lomustine, mitoxantrone, amsacrine, clofarabine, and/or fludarabine.

CNS: central nervous system. HMA: hypomethylating agents. TKD: tyrosine kinase domain.

### HMA treatment indication and modalities

Median time between diagnosis and HMA therapy initiation was 215 days, with a very wide range, from 1 day to more than 14 years for a patient who experienced a late CBF-AML relapse. HMA were used as frontline treatment for 20 patients (41%) with a median delay before treatment introduction of 16 days (range 1–91). While most of these patients were treated with HMAs because of their age (median age 74.5 years in this group), four patients under 65 years of age received HMAs as first-line treatment because of severe comorbidities contraindicating intensive chemotherapy (severe obesity, ischemic heart disease, septic shock), or because of patient choice. On the other hand, 29 patients (59%) received HMAs as second-line or more (after failure of intensive chemotherapy, for R/R CBF-AML), with a median time from diagnosis to HMA treatment of 476 days (range 104–5393) (Table 1). This group included 24 patients with cytologic CBF-AML relapse and 5 patients with molecular relapse or progression.

A total of 344 cycles of HMA were analyzed. Forty-six patients received subcutaneous injections of azacitidine and 3 received decitabine intravenously. The median number of HMA cycles administered was 5 (range 1–36), with a median of respectively 6 (range 1–36) and 4 cycles (range 1–20) for patients who received HMA therapy as frontline treatment and for R/R CBF-AML. Most of patients received azacitidine at standard dose of 75 mg/m^2^/d for 7 days (consecutive or not), except for one patient who received reduced doses of 60 mg/m^2^/d from the 3rd cycle due to hematological toxicity. All administered courses of decitabine were at 20 mg/m^2^/d for 5 days. As expected, for patients who received at least two cycles, median delay between consecutive HMA cycles was 28 days.

In most cases, treatment was stopped for progressive disease (*n* = 34; 69%). Other reasons for HMA discontinuation were patient’s choice (*n* = 4; 8%), complete response with undetectable MRD (*n* = 3; 6%), switch for intensive therapy, including allogeneic stem cell transplant (*n* = 3; 6%), non-hematologic toxicity (*n* = 2; 4%), death (*n* = 2; 4%), and a prostate cancer progression (*n* = 1; 2%).

### Effectiveness of HMA treatment

Eight patients were excluded from this analysis as their therapeutic response was not assessable according to the ELN 2017 criteria. Of the remaining 41 patients, 20 responded to HMA treatment, giving an overall response rate (ORR) of 49%. Best response was CR_MRD-_ in 4 patients, CR in 10 patients, and CRi in 6 patients. Median time to response was 112 days (range 28–183), corresponding to 4 HMA cycles (range 1–6). According to HMA indication, the ORR was respectively 69% (11/16) and 36% (9/25) for patients who received HMA as frontline therapy or for R/R CBF-AML (Chi-squared test, *p* = 0.041). For the 21 non-responding patients, best response was stable disease (*n* = 7), progressive disease (*n* = 10), treatment failure (*n* = 2), hematologic relapse (*n* = 1) or molecular progression (*n* = 1). Table 2 compares clinical, biological and HMA treatment characteristics for responders and non-responders. Different characteristics between responders and non-responders were number of therapeutic lines prior HMA introduction and the total number of received HMA cycles.

**Table 2:** Comparison of clinical and biological characteristics between responders and non-responders to HMA therapy

	Responders (*n* = 20)	Non-responders (*n* = 21)	*p*-value
Age (years)	56.5 [30—86]	63 [23—85]	ns
Gender (female)	10 (50%)	10 (48%)	ns
Performance status:			ns
• PS 0 or 1 • PS 2 or 3	17 (85%) 3 (15%)	14 (67%) 7 (33%)	
CBF-AML subtype:			ns
• t(8;21) and/or *RUNX1*::*RUNX1T1* transcript • inv(16) or t(16;16) and/or *CBFB*::*MYH11* transcript	9 (45%) 11 (55%)	8 (38%) 13 (62%)	
Secondary CBF-AML	3 (15%)	7 (33%)	ns
Extra-medullar and/or CNS involvement	4 (20%)	4 (19%)	ns
Biological data:			
• Bone marrow blasts (%) • Hemoglobin (g/L) • White blood cells (G/L) • Neutrophils (G/L) • Platelets (G/L)	25 [1—81] 104 [64—142] 4.1 [1.1—190.3] 1.5 [0.1—11.3] 63 [9—227]	33.5 [2—88] 102 [74—137] 4.0 [0.8—42.7] 2.0 [0.0—22.0] 70 [7—214]	ns ns ns ns ns
Additional cytogenetic abnormalities	10 (50%)	11 (52%)	ns
Number of prior therapeutic lines	0 [0–2]	2 [0–4]	0.045
CBF-AML status at HMA initiation:			ns
• Diagnosis • Hematologic relapse • Molecular relapse or progression	11 (55%) 6 (30%) 3 (15%)	5 (24%) 14 (67%) 2 (10%)	
HMA type:			ns
• Azacitidine • Decitabine	17 (85%) 3 (15%)	21 (100%) 0	
Number of received cures of HMA	8.5 [1–36]	3 [1–12]	< 0.001

Data are median [range] or number (%). “Responders” included patients with complete response (CR_MRD-_, CR, or CRi).

CNS: central nervous system.

At HMA initiation, 32 patients were RBC and/or platelet transfusion-dependent (65%). For RBC, 32 patients (65%) were transfusion-dependent prior to HMA introduction, of whom 9 became transfusion-independent (28%). The median number of HMA courses before RBC transfusion-independency was 3 (range 2–5). For platelets, 27 patients (55%) were transfusion-dependent prior to HMA introduction, of whom 9 became transfusion-independent (33%). The median number of HMA courses before platelet transfusion-independency was 3 (range 3–4). Twenty-one patients had a grade 3 or 4 neutropenia (43%) at HMA initiation, and hematological-improvement for neutrophils was noticed for 10 patients (48%).

### Survival analysis

**Fig. 1:**
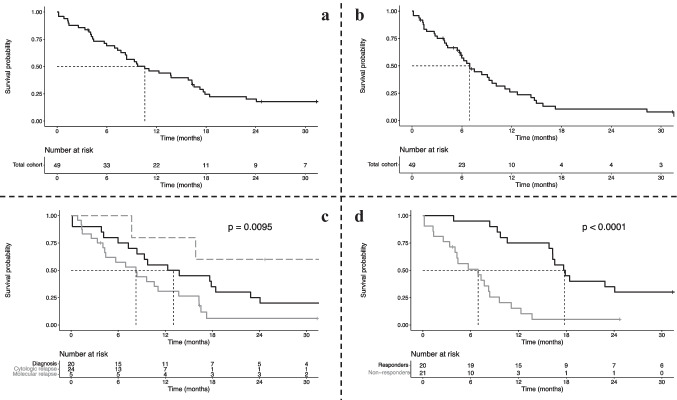
Kaplan–Meier survival curves. **a**. OS survival curve for the total cohort. **b**. EFS survival curve for the total cohort. **c**. OS survival curve according to AML status at HMA onset: diagnosis (black line), hematologic relapse (grey line) or molecular relapse or progression (dashline). **d**. OS survival curve for HMA responders (black line) or non-responders (grey line)

At the end of follow-up, 7 patients were still alive (14%). After a median follow-up of 72.3 months (95%CI: 31.3-NR), median overall survival (OS) was 10.6 months (95%CI: 8.3–16.3) for the total cohort, with a one-year OS at 46.0% (95%CI: 33.9–62.5) (Fig. 1a). Median EFS was 6.9 months (95%CI: 5.7–10.1), with a 1-year EFS of 26.2% (95%CI: 15.6–44.1) (Fig. 1b). Median overall survival when HMA were used at diagnosis was 13.0 months (95%CI: 8.4–24.1), and 9.6 months (95%CI: 5.7–16.3) when used as second-line therapy or more (log-rank test, *p* = 0.6) (*Supplemental *Fig. 1). Nevertheless, OS was significantly different according to CBF-AML status at HMA introduction with a 1-year OS of 55.0% (95%CI: 37.0–81.8) for diagnosis, 30.9% (95%CI: 16.7–57.0) for hematologic relapse and 80.0% (95%CI: 51.6–100) for molecular relapse or progression (log-rank test, *p* = 0.0095) (Fig. 1c). Moreover, response to HMA treatment significantly improved OS compared to non-responding patients, with a respectively one-year OS of 75.0% (95%CI: 58.2–96.6) and 15.3% (95%CI: 5.4–43.3) (log-rank test, *p* < 0.0001) (Fig. 1d).

**Fig. 2:**
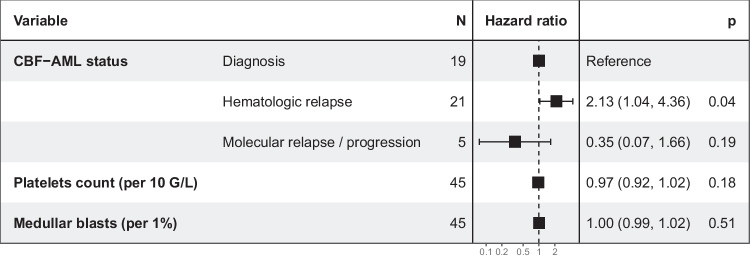
Multivariate Cox regression analysis on baseline parameters for overall survival

Among baseline parameters, CBF-AML status at HMA initiation (diagnosis *vs*. hematological relapse *vs*. molecular relapse or progression), platelets count (per 10 G/L) and medullar blast percentage were associated with OS in univariate analysis (p < 0.1) (*Supplemental *Table 1). These parameters were therefore included in multivariate analysis, which demonstrated that hematological relapse status was significantly associated with shorter OS for patients treated with HMA (HR: 2.13; 95%CI: 1.04–4.36; *p* = 0.038) (Fig. 2).

### Safety of HMA treatment

As expected, most frequently reported adverse events (AEs) were cytopenia. Forty-two patients (86%) experienced at least one episode of grade 3 or 4 cytopenia during HMA courses. Grade 3 or 4 neutropenia, anemia and thrombocytopenia occurred in respectively 67%, 69% and 65% of patients. Three and 5 patients became respectively RBC and platelets transfusion-dependent during HMA treatment. At least one episode of febrile neutropenia was reported in 24 patients (49%), of which three died of septic shock. Most of the clinically documented infections involved lung, ENT (Ear, Nose, Throat) or skin. Bleeding symptoms were noted in 12 patients (24%). Most of them were mild skin or mucosal hemorrhages, but one patient died from cerebral bleeding.

Declared non-hematological AEs were mostly mild. Most common were skin reaction at injection site (49%), nausea and/or vomiting (27%), diarrhea (8%) and constipation (8%). Other non-hematological AEs occurred in less than 5% of patients. It should be noted that two patients died after the onset of non-febrile dyspnea of unknow cause. Both of them were receiving their first course of HMA for newly diagnosed CBF-AML. Pulmonary leukostasis and tumor lysis syndrome were discarded in both cases.

## Discussion

Even if CBF-AML are classified as favorable prognosis when treated with intensive chemotherapy, reported 5-year relapse rate remains high, with no consensus about the best second-line therapy. Hypomethylating agents are an interesting therapeutic option for patients experiencing R/R AML [42, 43], or for frail patients who cannot handle intensive chemotherapy [44]. Nevertheless, CBF-AML patients were excluded from the phase III clinical trials evaluating the efficacy of azacitidine or decitabine for high-risk MDS or AML [28–32]. CBF-AML were also excluded from the recent QUAZAR AML-001 study which demonstrated that maintenance with oral azacitidine CC-486 prolongs OS and RFS in patients with AML in first remission after intensive chemotherapy who were not candidates for allogeneic stem cell transplant [45]. In this retrospective study, we report an overall response rate of 49% for CBF-AML patients treated with azacitidine or decitabine with a median delay to response of 4 HMA cycles. Median overall survival when HMA were used upfront was 13.0 months (95%CI: 8.4–24.1), and 9.6 months (95%CI: 5.7–16.3) when used as second-line therapy or more for R/R CBF-AML. Response was significantly associated with improved OS with a 1-year OS of 75.0% for responders *vs*. 15.3% for non-responders (p < 0.0001). As expected, HMA toxicity was consistent with the well-known safety profile of HMA published in prospective studies in other AML subtypes [31, 46]. To our knowledge, this is the largest real-life cohort published about effectiveness of HMA in CBF-AML subgroup.

The epigenetic abnormalities and the effectiveness of HMA in the subset of CBF-AML has been widely suggested by pre-clinical studies, especially in AML with t(8;21). Indeed, it has been demonstrated that the aberrant fusion protein RUNX1::RUNX1T1 was able to recruit a transcriptional repressor complex including DNA methyltransferase 1 (DNMT1) and histone deacetylases (HDACs), leading to chromatin remodeling, silencing of several genes involved in normal hematopoiesis and differentiation blockage in CBF-AML with t(8;21) [8, 9, 47]. The in vitro exposition of leukemic cells expressing this fusion protein to DNMT inhibitors (such as decitabine or azacitidine) led to restoration of normal gene expression and cell differentiation, especially when combined with HDAC inhibitors. Moreover, mutations in epigenetic regulators, and especially genes involved in DNA methylation (such as *TET2* or *DNMT3A*), are significantly enriched in *RUNX1*::*RUNX1T1* CBF-AML, and play a key role in leukemogenesis process [48, 49]. A recent study even suggested that these mutations are associated with shorter OS [50]. On the other hand, very few epigenetic regulator genes were found mutated in CBF-AML with inv(16). Nevertheless, aberrant DNA methylation has also been described in this specific subtype of AML. A recent study demonstrated that the fusion protein CBFB::MYH11 impairs the normal interaction between DNMT3A and RUNX1, leading to the hypomethylation and the hyperexpression of genes involved in AML progression [51]. It has also been demonstrated that the promotor of another RUNX family gene, *RUNX3*, was frequently hypermethylated in CBF-AML with inv(16) [52]. Despite these data suggesting impaired epigenetic mechanisms in CBF-AML, very few were published about clinical HMA efficiency in this AML subgroup.

In a retrospective study about R/R CBF-AML, Khan et al.included 6 patients who received HMA as first salvage therapy [27]. They reported a CR rate of 16.7% (*n* = 1/6), which is consistent with the low ORR we reported (36%, *n* = 9/25). In their study, the median OS was shorter, but not statistically different from patients who received high dose chemotherapy as salvage treatment. This result might be due to the small number of patients treated with HMA. Moreover, three published studies examined the role of HMA maintenance after intensive chemotherapy. First, in a prospective study, Blum et al.reported results about 46 young adults with CBF-AML in CR1 who received decitabine maintenance after high-dose chemotherapy without ASCT [53]. One-year disease-free survival (DFS) was 80%, and did not statistically differ from reported DFS in non-CBF-AML patients. Senapati et al.reported in a prospective study that decitabine maintenance in CBF-AML patients in CR1 after intensive chemotherapy with persistent molecular disease led to complete molecular response in 52% of cases (*n* = 12/23) [54]. Nevertheless, even if the median molecular relapse free-survival in responders was 93.9 months, there was no difference in overall survival between responders and non-responders to HMA therapy. Finally, Ragon et al.reported retrospective results about 23 CBF-AML patients receiving azacitidine maintenance after chemotherapy [55]. The authors concluded that patients with low levels of MRD might benefit from HMA maintenance to prolong survival, especially for those who experienced MRD reduction after two cycles of HMA therapy. These studies and our suggest that HMA could be of therapeutic interest in the setting of CBF-AML.

Nevertheless, multiple bias and limits might be discussed about our study. First, the number of patients is low and the population heterogeneous, HMA treatment for CBF-AML remaining a rare situation in clinical practice. Among all the adult CBF-AML patients we screened, about 8% only received HMA treatment at any time during AML evolution, although we limited exclusion criteria to prevent selection bias. Secondly, retrospective studies imply a bias in data collection and analysis. To avoid declaration heterogeneity, we retrospectively re-assessed AML status at HMA initiation and response status after all HMA courses according to published recommendations [38], allowing a reproducible evaluation between all patients. Response was assessable after about one third of all HMA courses. Indeed, if clinical or blood evaluation may be sufficient to assess progressive disease, bone marrow aspiration is mandatory to reach a conclusion of complete or partial remission [38]. Therefore, because of the low proportion of patients who benefited from bone marrow evaluation, we might have underestimated the ORR. The toxicity assessment may also have been underestimated due to retrospective data collection, but it is unlikely that the safety profile of HMA is different in the CBF-AML subgroup compared to the others.

Finally, it has to be noted that HMA are not used as monotherapy anymore for AML treatment. The study VIALE-A demonstrated that association of azacitidine with BCL2 inhibitor venetoclax improves patients OS, leading to the recent approval of this combination for newly diagnosed AML in intensive chemotherapy ineligible patients [32]. Nevertheless, CBF-AML were also excluded from this study, and no clue is currently available for the efficacy of azacitidine + venetoclax combination in this AML subgroup, with the exception of one case report [56]. HMA may also be associated with other drugs whose anti-leukemic activity in CBF-AML have been suggested, such as gemtuzumab-ozogamicin [26, 57], or tyrosine-kinase inhibitors dasatinib [58, 59] or midostaurin [60].

## Conclusion

In conclusion, our study highlights that hypomethylating agents are a well-tolerated therapeutic option for R/R CBF-AML and for patients who cannot handle intensive chemotherapy. Although the efficacy of HMA appears similar in CBF-AML to that reported in other subtypes of AML, our results seem suboptimal in the context of this AML subset associated with a "favorable" prognosis. HMA efficacy might be improved if combined with other therapies such as BCL-2 inhibitor venetoclax, or tyrosine-kinase inhibitors in CBF-AML.

### Supplementary Information

Below is the link to the electronic supplementary material.

Supplementary file1 (PPT 60 KB)

Supplementary file2 (DOCX 16 KB)

## Data Availability

The datasets used and/or analysed during the current study are available from the corresponding author on reasonable request.

## Abbreviations

AEs: Adverse events
AML: Acute myeloid leukemia
ASCT: Allogeneic stem cell transplant
CBF: Core binding factor
CR: Complete remission
CRi: Complete remission with incomplete hematologic recovery
CR_MRD-_: Complete remission with undetectable measurable residual disease
DFS: Disease-free survival
ELN: European LeukemiaNet
EFS: Event-free survival
DNMT: DNA methyltransferases
HDAC: Histone deacetylases
HMA: Hypomethylating agent
MRD: Measurable residual disease
ORR: Overall response rate
OS: Overall survival
PR: Partial remission
RBC: Red blood cells
RFS: Relapse-free survival
RT-qPCR: Real-time quantitative polymerase chain reaction
R/R: Relapsed / refractory
