# Supplementary material for: Hypomethylating agent monotherapy in core binding factor acute myeloid leukemia: a French multicentric retrospective study
Source: Ann Hematol. 2024 Jan 26;103(3):759–69. doi: 10.1007/s00277-024-05623-0 (PMC10867066; doi:10.1007/s00277-024-05623-0)
Supplement: Supplementary file 1 — Supplementary file1 (PPT 60 KB) [file 277_2024_5623_MOESM1_ESM.ppt]

## Slide 1
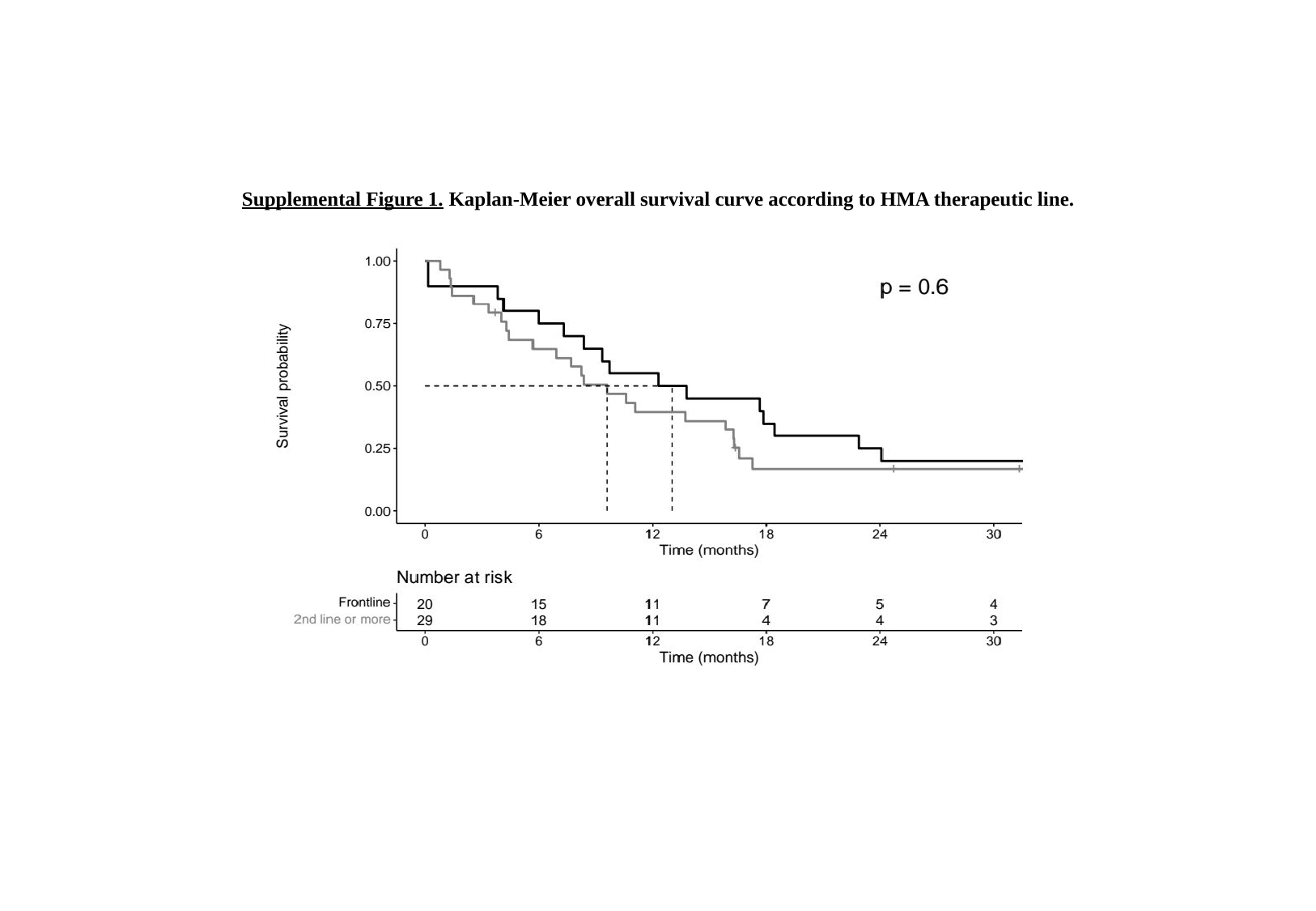

Supplemental Figure 1. Kaplan-Meier overall survival curve according to HMA therapeutic line.
