# Supplementary material for: Hypomethylating agent monotherapy in core binding factor acute myeloid leukemia: a French multicentric retrospective study
Source: Ann Hematol. 2024 Jan 26;103(3):759–69. doi: 10.1007/s00277-024-05623-0 (PMC10867066; doi:10.1007/s00277-024-05623-0)
Supplement: Supplementary file 2 — Supplementary file2 (DOCX 16 KB) [file 277_2024_5623_MOESM2_ESM.docx]

**Supplemental Table**

**Supplemental Table 1. Univariate Cox regression analysis on baseline parameters for overall survival**

|  | | HR (95%CI) | p-value |
| --- | --- | --- | --- |
| Age (per year) | | 1.01 [0.99 - 1.03] | 0.337 |
| Gender | Female | *reference* | |
|  | Male | 1.05 [0.57 - 1.94] | 0.874 |
| Performance  status | 0-1 | *reference* | |
|  | 2-3 | 1.48 [0.76 - 2.90] | 0.248 |
| AML status | Diagnosis | *reference* | |
|  | Hematological relapse | 1.90 [0.97 - 3.72] | 0.061* |
|  | Molecular relapse *or* progression | 0.27 [0.06 - 1.15] | 0.076* |
| CBF-AML type | Alpha | *reference* | |
|  | Beta | 0.71 [0.39 - 1.30] | 0.267 |
| De novo / secondary  AML status | *De novo* AML | *reference* | |
|  | Secondary AML | 1.52 [0.76 - 3.04] | 0.239 |
| Extra-medullar *and/or*  CNS involvement | No | *reference* | |
|  | Yes | 0.99 [0.41 - 2.36] | 0.974 |
| Hemoglobin (*per* 1 g/L) | | 1.00 [0.98 - 1.01] | 0.805 |
| Platelets count (*per* 10 G/L) | | 0.95 [0.91 - 1.00] | 0.070* |
| White blood cell count (*per* 1 G/L) | | 1.00 [0.99 - 1.01] | 0.641 |
| Absolute neutrophil count (*per* 1 G/L) | | 1.04 [0.94 - 1.14] | 0.452 |
| Medullar blast level (*per* 1%) | | 1.01 [1.00 - 1.02] | 0.084* |

* Parameters included for multivariate analysis (p < 0.2).

*HR: hazard ratio. CNS: central nervous system.*
